# MetaFun: unveiling sex-based differences in multiple transcriptomic studies through comprehensive functional meta-analysis

**DOI:** 10.1186/s13293-024-00640-0

**Published:** 2024-08-27

**Authors:** Pablo Malmierca-Merlo, Rubén Sánchez-Garcia, Rubén Grillo-Risco, Irene Pérez-Díez, José F. Català-Senent, María de la Iglesia-Vayá, Marta R. Hidalgo, Francisco Garcia-Garcia

**Affiliations:** 1 Computational Biomedicine Laboratory, Principe Felipe Research Center (CIPF), Eduardo Primo Yúfera Street, 3, 46012 Valencia, Spain; 2 Biomedical Imaging Unit FISABIO-CIPF, Fundación Para el Fomento de la Investigación Sanitaria y Biomédica de la Comunidad Valenciana, 46012 Valencia, Spain; 3 Department of Mathematics, Faculty of Mathematics, University of Valencia (UV), 46100 Burjassot-Valencia, Spain

**Keywords:** Systematic review, Meta-analysis, Web tool, RNA-sequencing, Functional profiling, Sex-based differences, Personalized medicine, FAIR data

## Abstract

**Background:**

While sex-based differences in various health scenarios have been thoroughly acknowledged in the literature, we lack sufficient tools and methods that allow for an in-depth analysis of sex as a variable in biomedical research. To fill this knowledge gap, we created MetaFun as an easy-to-use web-based tool to meta-analyze multiple transcriptomic datasets with a sex-based perspective to gain major statistical power and biological soundness.

**Description:**

MetaFun is a complete suite that allows the analysis of transcriptomics data and the exploration of the results at all levels, performing single-dataset exploratory analysis, differential gene expression, gene set functional enrichment, and finally, combining results in a functional meta-analysis. Which biological processes, molecular functions or cellular components are altered in a common pattern in different transcriptomic studies when comparing male and female patients? This and other biological questions of interest can be answered with the use of MetaFun. This tool is available at https://bioinfo.cipf.es/metafun while additional help can be found at https://gitlab.com/ubb-cipf/metafunweb/-/wikis/Summary.

**Conclusions:**

Overall, Metafun is the first open-access web-based tool to identify consensus biological functions across multiple transcriptomic datasets, helping to elucidate sex differences in numerous diseases. Its use will facilitate the generation of novel biological knowledge that can be used in the research and application of Personalized Medicine considering the sex of patients.

## Background

Sex-based differences in different health scenarios have been thoroughly acknowledged in the literature [1, 2]; however, this variable remains incompletely analyzed in many cases. Studies often neglect sex as a variable when considering the experimental design of studies, leading to experiments with samples of just one sex in extreme cases. As a result, the underlying mechanisms behind sex-based differences in many diseases and disorders remain incompletely established.

Fortunately, the scientific community has worked to significantly improve this situation in recent times, and researchers have begun to include the sex perspective in their research; however, a vast amount of generated data currently stored in public databases [such as Gene Expression Omnibus (GEO) [3] or NCI’s Genomic Data Commons (GDC) [4]] remains unanalyzed from this perspective. The information in these databases represents a powerful resource that must be considered.

When exploiting these resources with a particular objective, multiple studies dealing with similar scientific questions can provide different and often contradictory results. No one study is likely to provide a definitive answer; therefore, integrating all datasets into a single analysis may provide the means to understand the results. Designed for this purpose, meta-analysis is a statistical methodology that considers the relative importance of multiple studies upon combining them into a single integrated analysis and extracts results based on the entirety of the evidence/samples [5–7]. Unfortunately, applying advanced statistical techniques such as meta-analysis often remains out of reach for researchers aiming to analyze their data in a straightforward manner.

We designed the “MetaFun” tool to simplify the analytical process and facilitate the application of functional meta-analysis to researchers working with multiple transcriptomic datasets. Meta-analysis approaches can analyze datasets from perspectives such as sex and combine datasets to gain significant statistical power and soundness. MetaFun is a complete suite that allows the analysis of transcriptomics data and the exploration of the results at all levels, performing single-dataset exploratory analysis, differential gene expression, gene set functional enrichment, and finally, combining results in a functional meta-analysis.

There are currently other suitable tools that allow meta-analysis techniques to be applied to omics data, such as MetaGenyo (https://metagenyo.genyo.es/) for the Meta-Analysis of Genetic Association Studies, or ImaGEO (https://imageo.genyo.es/) for the Integrative Meta-Analysis of GEO Data. Compared to these tools, we present Metafun as an powerful alternative due to a double potential: on the one hand, and to our knowledge, it is the only web tool capable of integrating biological functions (while tools usually focus on the meta-analysis of genes or variants). Another important aspect is that Metafun is currently the only tool that can evaluate the different functional profiles, considering sex information. Both features provide a high-performance profiling tool for integrative user analyses.

## Construction and content

The MetaFun tool is available at https://bioinfo.cipf.es/metafun while additional help can be found at https://gitlab.com/ubb-cipf/metafunweb/-/wikis/Summary and https://gitlab.com/ubb-cipf/metafunweb/-/wikis/Study-Case. The minimum requisites the user must meet is a modern web browser as well as an internet connection.

### Input data and experimental design

**Fig. 1:**
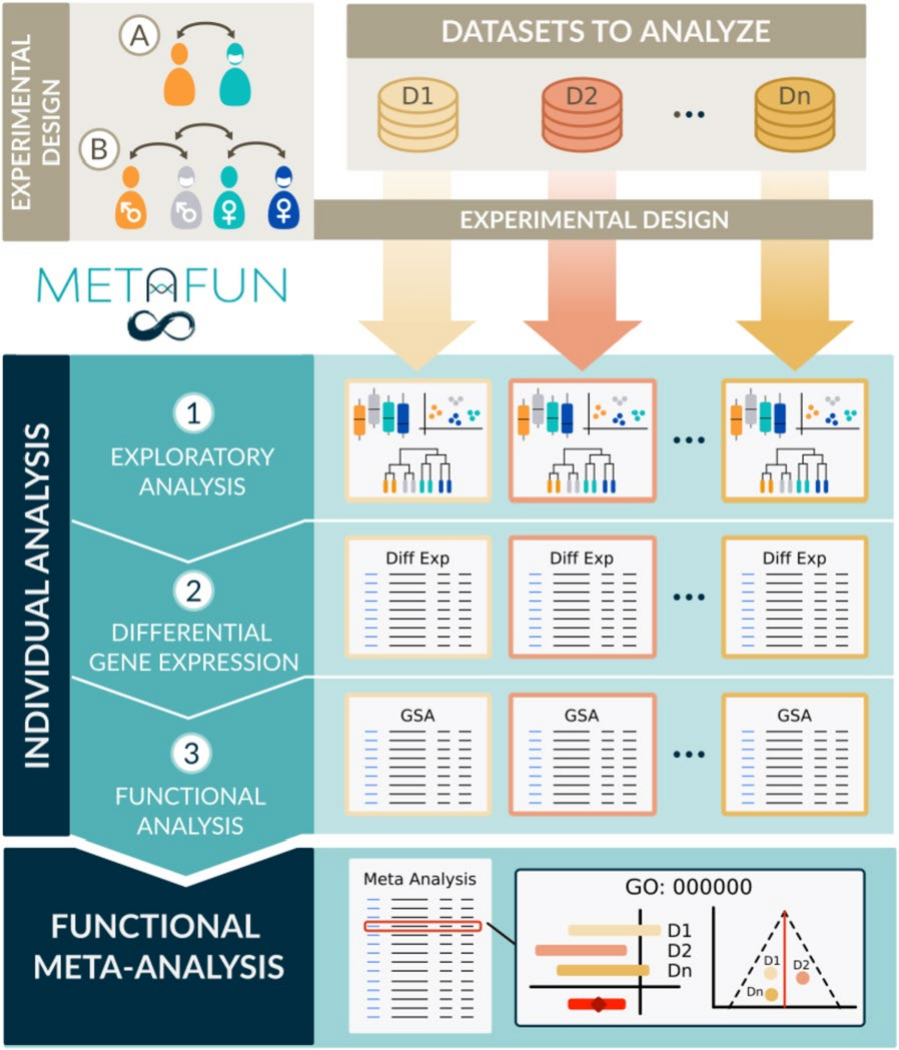
MetaFun pipeline. First, datasets and experimental designs are uploaded as CSV and TSV files. Available comparisons include **A** a classical comparison—*Case vs. Control*—and **B** a sex-perspective comparison—(*Female case* vs. *Female control*) vs. (*Male case* vs. *Male control*). Single experiment analyses include 1) exploratory analysis, 2) differential gene expression, and 3) functional analysis performed on each dataset. Finally, the results are integrated into a functional meta-analysis. The MetaFun tool allows users to explore all results generated during the process

MetaFun takes a set of at least two CSV expression files and two TSV experimental design files as inputs. CSV expression files must include normalized transcriptomics data from comparable studies with assimilable experimental groups. Columns must contain the study samples, while rows must contain analyzed genes as their Entrez Gene ID. The first row contains sample names. TSV experimental files define the class to which each sample of the study belongs by including at least two columns: sample names and the class to which they belong. Column names in the CSV expression files must match row names in the corresponding TSV experimental file. Accepted reference organisms are (for the moment) humans (*Homo sapiens*), mice (*Mus musculus*), and rats (*Rattus norvegicus*). Analyses can be made with respect to a comparison that must apply to all datasets. Options include the classical comparison—*Case* vs. *Control* (Fig. 1A)—or a sex-perspective comparison—(*Male case* vs. *Male control*) vs. (*Female case* vs. *Female control*) (Fig. 1B)—in which the effect under study is compared between sexes. This classical comparison: *Case vs. Control*, can be used to compare any two groups. It is also possible to assess the effect of a specific variable that stratifies the case and control groups by evaluating the results in 2 jobs. For example, it would be possible to know the effect of age when comparing “older patients vs. older controls” vs. “young patients vs. young controls”. The results obtained could be compared, identifying biological functions common to both comparisons as well as specific ones.

### Single dataset analyses

After the selection of the studies and experimental design, MetaFun analyzes each dataset separately with an individual analysis consisting of:

- an exploratory analysis including boxplots, PCA, and cluster plots using the *plotly* library [8]
- a differential gene expression analysis using the *limma* package [9]
- a gene set enrichment analysis (GSA) [10] based on gene ontology (GO) [11] from the *mdgsa* package [12].

Figures and tables from these analyses can be explored and downloaded from the *Results* area once the job has been completed. Links to NCBI [13] and QuickGO [14] databases are present to detail the results.

### Functional meta-analysis

MetaFun combines the gene set functional enrichments from all datasets into a meta-analysis with the same experimental design using the *metafor* package [15]. Forest and funnel plots are generated utilizing the *plotly.js* library [8]. Figures and tables from this meta-analysis are interactive and may be explored and downloaded from the *Results* area once the job has been completed.

### Implementation

The MetaFun back end was written using Java and R and is supported by the non-relational database *MongoDB* [16], which stores the files, users, and job information. The front end was developed using the *Angular* framework [17]. All graphics generated in this web tool were implemented with *plotly* [8] except for the exploratory analysis cluster plot, which uses the *ggplot2* R package [18].

### Study cases

As an example, MetaFun includes two sets of pre-selected study cases, one for each accepted species: human and rat. The study cases can be executed directly from the web tool, allowing the tool’s functionalities to be easily explored. The human study case includes nine studies from lung cancer patients [6].

## Utility and discussion

### Web tool overview

The web tool can be used with registered or anonymous users. Registered users will keep their data and jobs stored from one session to the other, while data and jobs from anonymous users will not be saved after leaving the session. The general design of the web tool includes an upper right menu with basic tool functionalities, a left side panel with specific submenus, and a central panel from which to interact with the web. Users are directed to a form launching a new job after logging in, which can be otherwise accessed through the *New Analysis* button in the top right menu. The *New Analysis* form passes through a series of steps, asking for information that has to be completed and allows for a new meta-analysis. After the launch and execution, the job will be listed in the jobs area, which can be accessed through the *My jobs* button located in the top right menu. All created jobs are listed and can be accessed through the left side submenu to visualize results. Users can access their personal area through the top right panel using a button carrying their username. Each user’s personal area includes a browser for folders and information regarding all launched jobs. The user's personal area submenu supports a series of actions related to personal settings and deleting options. The top right panel also includes an exit icon button that logs users out and a question mark icon that opens documentation pertinent to the web tool (accessible through https://gitlab.com/ubb-cipf/metafunweb/-/wikis/Summary).

## Input data

All datasets in the same meta-analysis should be comparable, including similar experimental designs and individuals with similar conditions. At least two datasets must be included in a meta-analysis. Input data consists of one expression matrix and one experimental design file for each dataset in the meta-analysis. Furthermore, we suggest that for each experimental group there should be a minimum of 3 samples in each experimental group. Although the statistical power of functional meta-analysis is greater than that of functional analysis of individual studies, it is advisable to take these considerations into account, especially when you have sex-based subgroups, where sample sizes are usually smaller.

The expression matrix must have been normalized, with samples in columns and Entrez ID genes in rows. The experimental design file must indicate the original group to which each sample belongs, with samples in rows and groupings in columns. More than one grouping per file is accepted, placing each grouping in a different column (for instance, the first column could be sex, the second column experimental conditions, and the third column disease grade). However, only one grouping at a time will be used in a meta-analysis.

Data from different technologies (RNA-Seq, microarray...) can be used as long as the input data format meets the requirements described in Metafun.

## Launching a meta-analysis

The *New Analysis* button in the top right menu directs users to a form that launches a new meta-analysis. The first tab of the form—labeled *Files*—includes a browser for user files and allows users to upload and manage the datasets to analyze. The *Options* tab allows users to specify the *Effect Model* to *random* or *fixed*, to select the reference organism among *Homo sapiens*, and *Rattus norvegicus*, to define the GO ontologies to analyze (*Biological Process*, *Molecular Function*, and *Cellular Component*), and whether to propagate the annotation. A brief description accompanies each option to help users in their decisions. The *Studies* tab is used to select the studies to meta-analyze and the experimental design. Depending on the case, selections are made by dragging files from the right panel entitled *My Files* to the columns *Expression* or *Experimental Design*. Matched studies and experimental design must be placed in the same row, verifying their compatibility by checking sample names in the expression and experimental design files. Users can specify the comparison to perform in the *Comparison* tab. Two different options are currently available: the classical *Case vs. Control*, which compares the effect of a variable, and a sex-based comparison—*(Case Female *vs.* Control Female) *vs.* (Case Male vs. Control Male)* —which compares the effect of a variable in females with respect to the effect in males. In the second case, significant results refer to differential effects between males and females and may not coincide with results from the first comparison. After selecting the comparison, users must indicate which study samples are included in each canonical compared group (*Case*, *Control*, *Case Female*, etc.) by assigning one of the classes in the experimental design of each study to these canonical groups. Finally, the *Launch* tab contains a summary of the defined meta-analysis, which may be launched through the *Launch job* button after the name assignment.

## Analysis summary

After the execution of the job and its selection in the left side panel of the *My Jobs* panel, the *Analysis summary* tab will show a summary of the main results, which include:

- selected analysis options—name, comparison, effect model, functional profile, and reference organism
- a table and an interactive barplot describing the number of samples per dataset and per group
- a table describing the number of differentially expressed genes in each dataset—per column, the studies, total number of analyzed genes, total number of significantly affected genes, number of significantly upregulated genes, and number of significantly downregulated genes
- a table including the same columns describing the number of significant functional profile items in each dataset—either enriched functions
- a table including the same columns describing the number of significant functional terms in each ontology—BP for Biological Process, MF for Molecular Functional, and CC for Cellular Component—of the meta-analysis

## Exploratory analysis

The *Exploratory analysis* tab contains the figures from the unsupervised exploratory analysis performed on each dataset in the meta-analysis. This analysis includes a boxplot representation of the expression of the samples, a clustering of the samples, and a principal components analysis (PCA) plot representing the first two components of the PCA. All samples are colored by the experimental design selected in the meta-analysis.

## Differential expression

The differential expression analysis is performed with the *limma* library [9], applying *lmFit, contrast.fit,* and *eBayes* functions while considering whether samples are paired or unpaired. Results will be displayed as a table in the *Differential expression* tab of the job. The table shows the Entrez ID, Gene Name, the logarithm 2 of the fold-change (logFC), test statistic, raw p-value, and Bonferroni-Holm [19] adjusted p-value of each analyzed feature. The raw p-value initially orders the table, but buttons on column names allow users to order the table differently. Links from the Entrez ID column direct to the NCBI gene database of the specific gene. Different tools allow users to search, download, and filter the table by a maximum p-value.

## Gene set analysis

The functional analysis consists of a GSA [10] based on the BP, MF, and CC ontologies from GO [11] defined by users. The pipeline, performed with the *mdgsa* library [12], splits the ontologies, propagates the annotation (if indicated), filters too generic (more than 500 annotated genes) or too specific (less than ten annotated genes) annotations, transforms the p-value into an index, and performs the corresponding comparisons. Results will be displayed as a table in the *GSA* tab of the job. Three subtabs on the top right of the table separately show the results for the three different ontologies. For each ontology, the table shows the GO ID, GO term, the logarithm of the odds-ratio (LOR), raw p-value, Bonferroni-Holm adjusted p-value, and the number of genes included in each analyzed feature. The raw p-value initially orders the table, and buttons on column names allow users to order the table differently. Links from the GO ID column direct to the QuickGO [14] entry of the specific term. Different tools allow users to search, download, and filter the table by a maximum p-value.

Functional meta-analysis integrates the results of the functional analysis and is performed using the rma function of the metafor package [15]. Specifically in Metafun, the rma function is applied to each biological function, performing a meta-analysis by combining the level of overrepresentation (LOR) of that function with the level of overrepresentation (LOR) of that function.

## Meta-analysis

The functional meta-analysis integrates the functional analysis results and is performed using the *rma* function of the *metafor* package [15]. This function allows the selection of different effect measures for meta-analysis (e.g., log risk ratios, log odds ratios, mean differences,...), as well as a wide variety of meta-analysis parameters and methods. Specifically in Metafun, the rma function is applied to each biological function, where a meta-analysis combines the level of overrepresentation (LOR) of that function in different studies. Two methods have been implemented to perform meta-analyses: the fixed effects models (FE) and the random effects models (DL DerSimonian & Laird; HS Schmidt & Hunter; Hedges, HE) [15]. The fixed effect model has been designed for similar studies (i.e., with the same technology, platform, and at similar times), while the random effect model allows for more significant variability. The random-effects model is often the most appropriate strategy when multiple datasets from different studies are available. In this case, a combined measure of the level of over-representation of a function is obtained by considering the variability in each of the studies. In this way, those studies with greater variability will contribute a lower weight in the set, while those studies with less variability and therefore more robust, will contribute a greater weight in the calculation of the level of overrepresentation of the biological function evaluated. Results will be displayed as a table in the *Meta-analysis* tab of the job. The table shows the GO ID, GO term, LOR, confidence interval of the LOR, raw p-value, and Bonferroni-Holm [19] adjusted p-value of each analyzed feature. The raw p-value initially orders the table, and buttons on column names allow users to order the table differently. Links from the GO ID column direct to the QuickGO [14] entry of the specific term. Different tools allow users to search, download, and filter the table by a maximum p-value.

## Strengths and limitations

Exploring sex bias can significantly improve biological and clinical outcomes in female and male patients in numerous human diseases. In the Metafun tool, we have implemented a strategy that allows for the evaluation and integration of several datasets, with the possibility of including sex information.

Most of the limitations associated with the use of Metafun are linked to the datasets selected for integration. It is therefore important that users take into account a number of recommendations: (a) if the data come from different platforms, technologies... the random-effects meta-analysis method will be more appropriate than the fixed-effects method; (b) the minimum sample size in each of the experimental groups should be3 samples. A larger sample size will improve the statistical power of our analysis, and a smaller size will make it more difficult to detect a possible biological signal; (c) in the same vein, if in our study we want to evaluate sex differences in a given disease, it is balance in the number of samples of male and female subjects to avoid possible biases in the results. It is preferable not to include data sets that do not present a balance in the samples of both sexes; (d) statistically significant results should be reviewed by means of forest plot and funnel plot to ensure their relevance and biological coherence in the set of integrated studies.

The application of this functional meta-analysis strategy provides a set of biological functions where we have identified a robust biological signal, across the set of data sets evaluated. We strongly encourage users to perform, whenever possible, the corresponding experimental validation to confirm the biological relevance of these results. There are multiple ways to carry out this experimental validation (confirmation of gene expression levels where there is a higher activation of the selected functions, functional validations...). All these approaches will increase the relevance of the results and improve their biological understanding.

Web tools require continuous development and improvement. Therefore, we have defined a future work plan for the next version of Metafun that will include: (a) the possibility of performing functional meta-analysis also on KEGG or REACTOME pathways; (b) extending the meta-analysis methods on the strategy used by the HiPathia tool for the evaluation of differential activation profiles; (c) direct selection of data sets from the GEO repository; (d) the option to incorporate in the differential expression analysis those control variables that may generate specific variability, such as age, disease stage of disease, etc.; (e) extending the stratified analysis scenarios; (f) extension of the stratified analysis scenarios.

## Study case

The following case describes the potential use of MetaFun in characterizing sex-based differences in lung adenocarcinoma. The results obtained were published in [6].

### Input data:

Each study requires two files: one with expression data and a second with the description of the experimental groups to which each sample belongs, indicating the sex of the participant.

The files corresponding to this use case can be downloaded at the following link—https://gitlab.com/ubb-cipf/metafunpipeline/-/blob/master/Homo%20Sapiens%20(Adenocarcinoma).zip

### Four simple steps to launch the meta-analysis job:


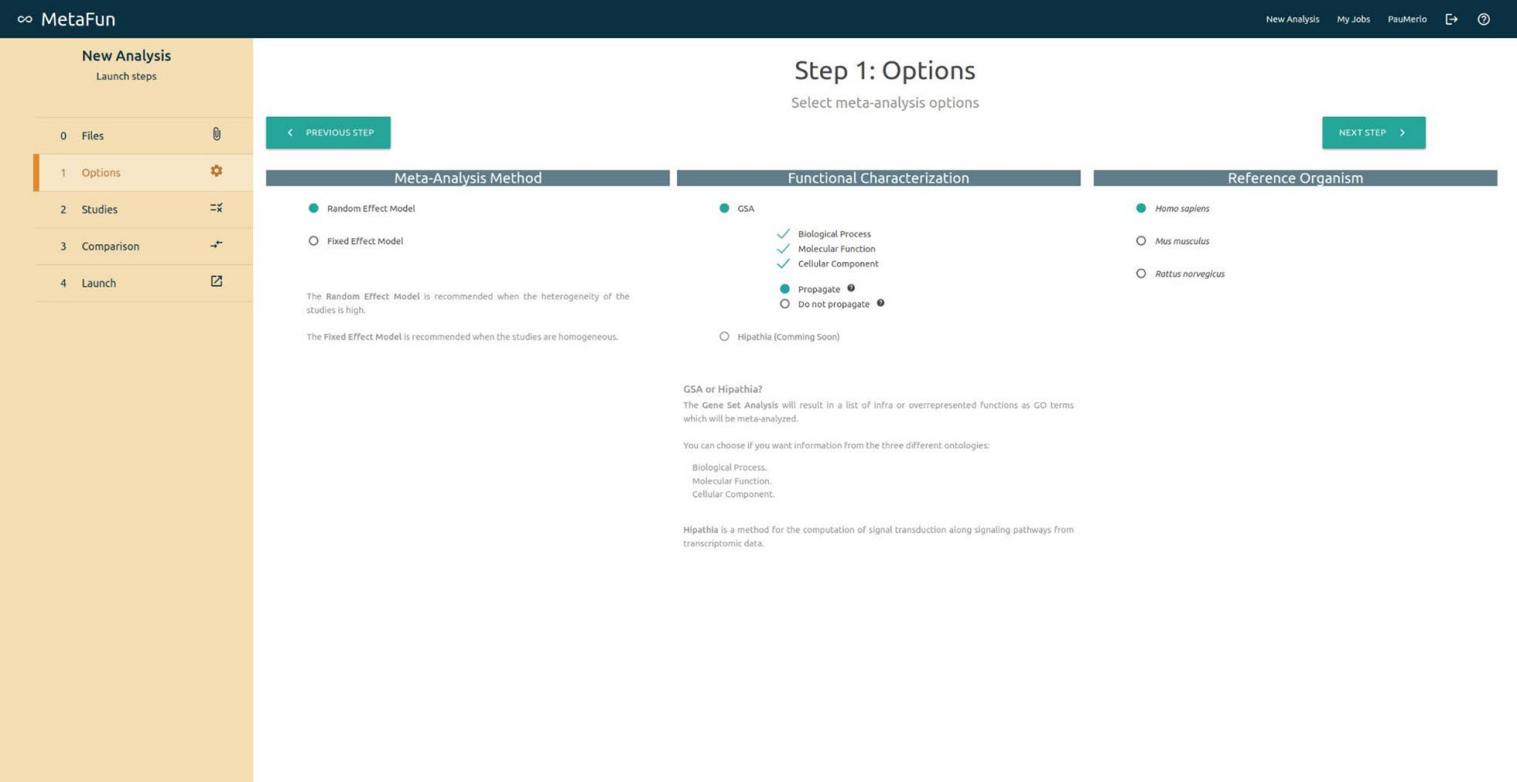



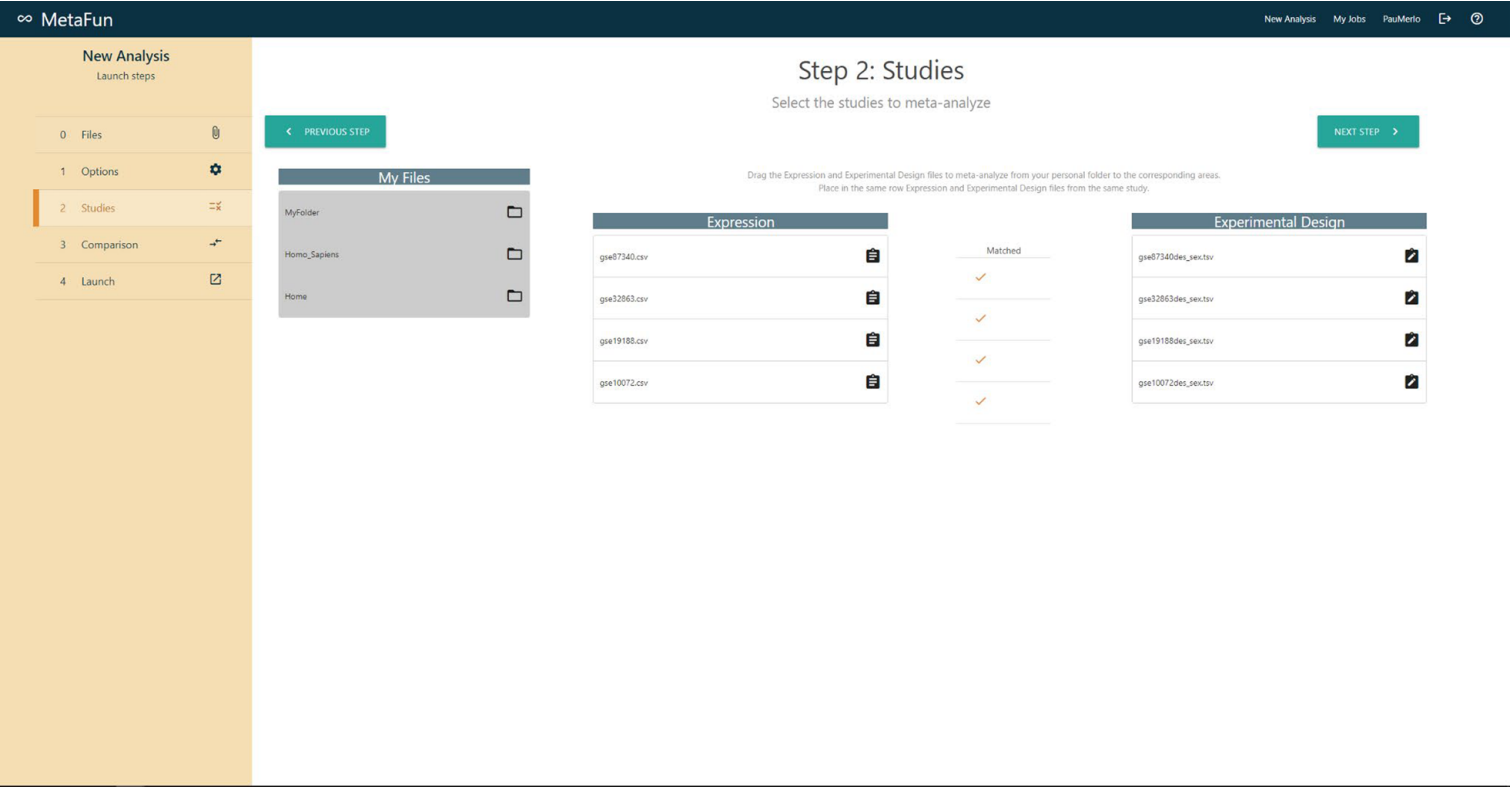



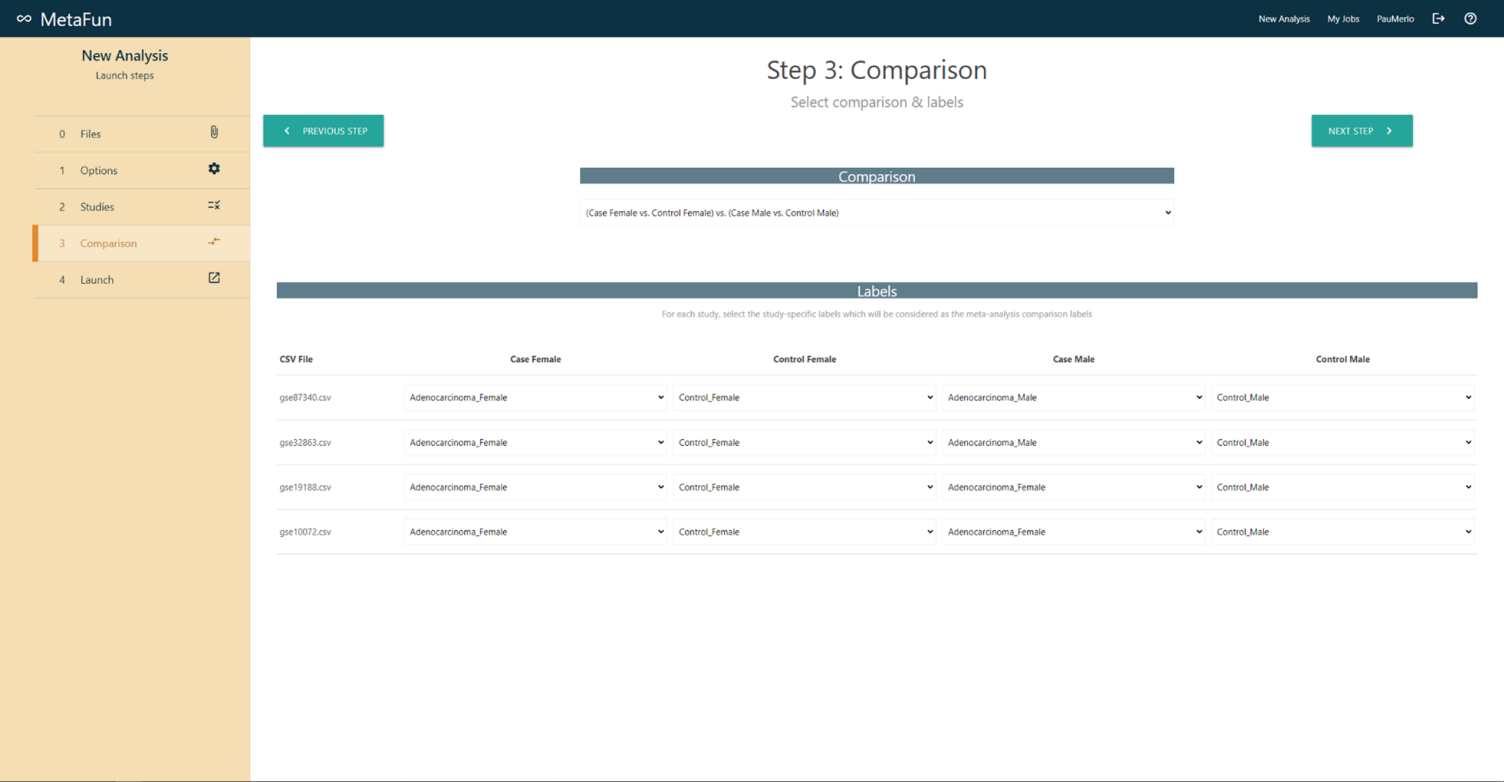



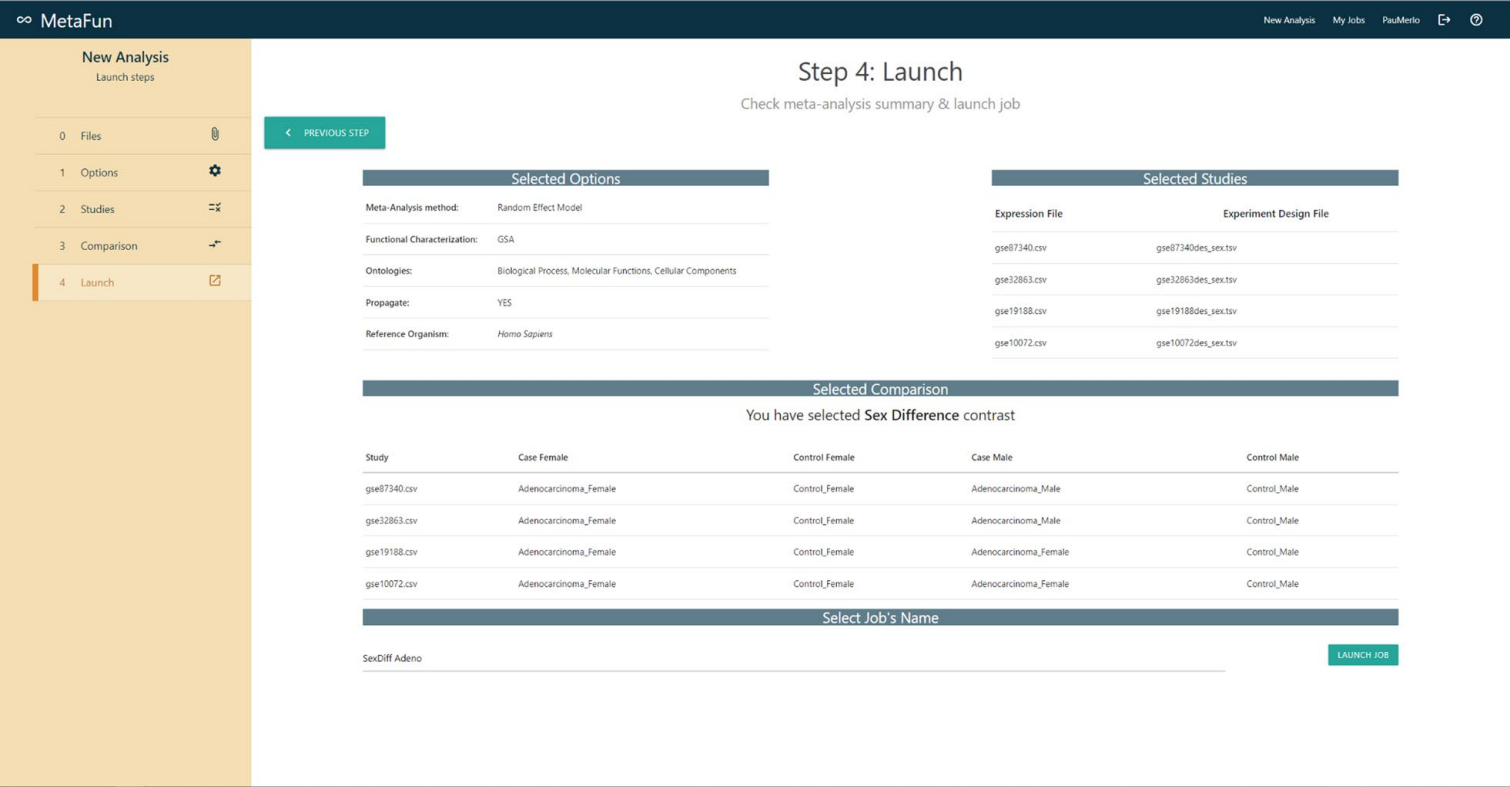


### Results:

Below, we display the results generated by MetaFun in this use case for each of the sections described above (1. Analysis Summary, 2. Exploratory Analysis, 3. Differential Expression, 4. Gene Set Analysis, 5. Meta-analysis),

#### 1. Analysis summary

A summary of the results at the distinct stages of the bioinformatics analysis strategy:
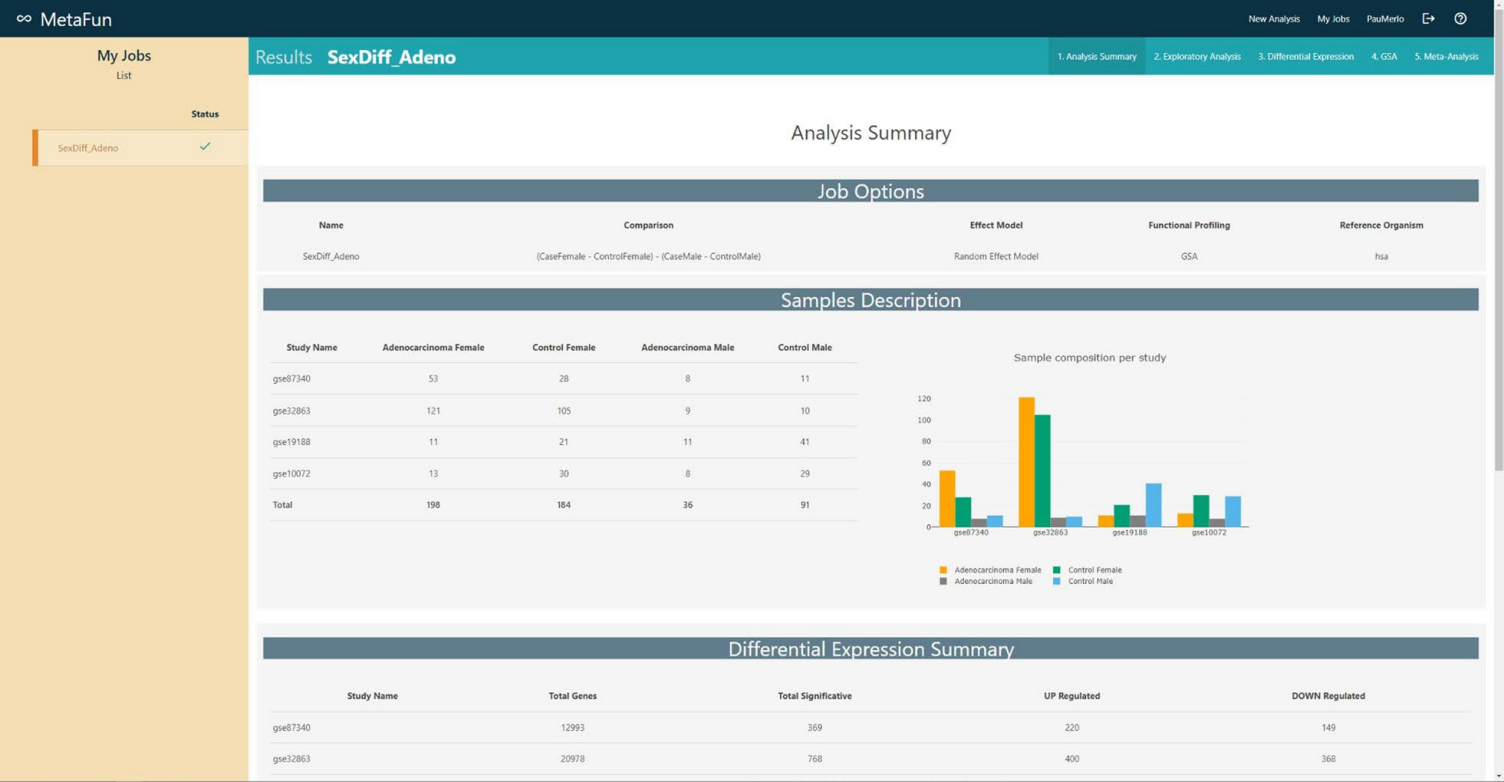



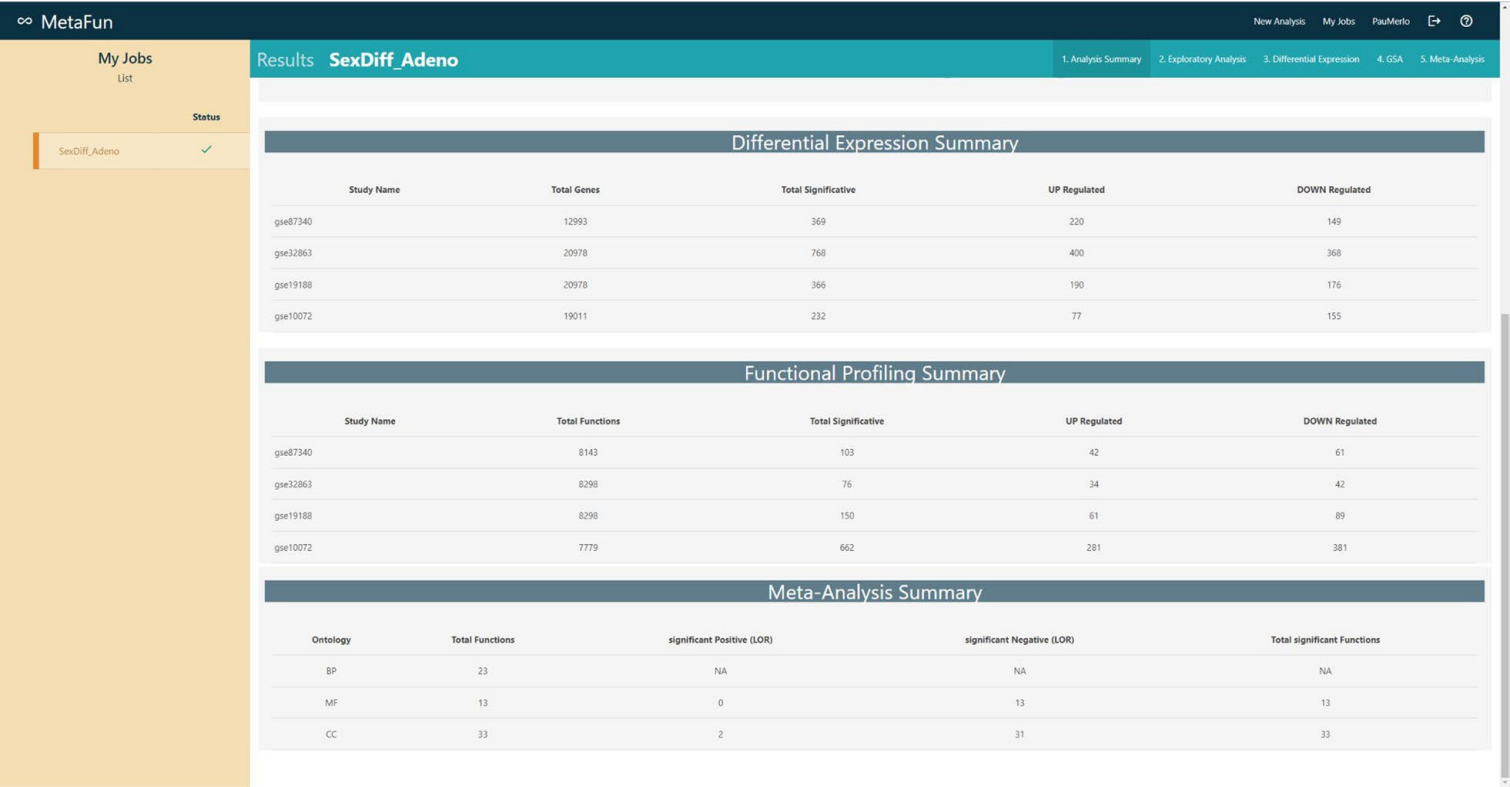


#### 2. Exploratory analysis

PCA, clustering, and boxplots are used to explore the expression levels of each of the samples in the selected studies:
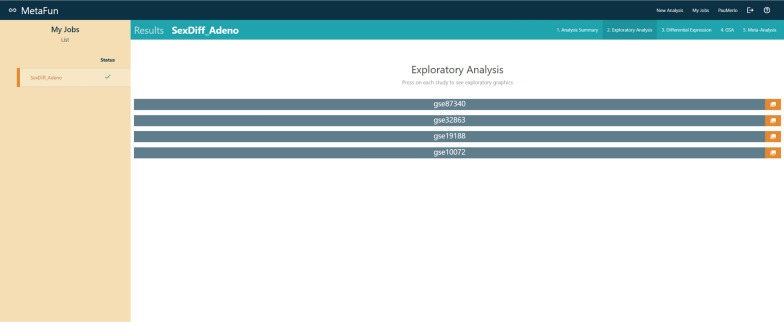



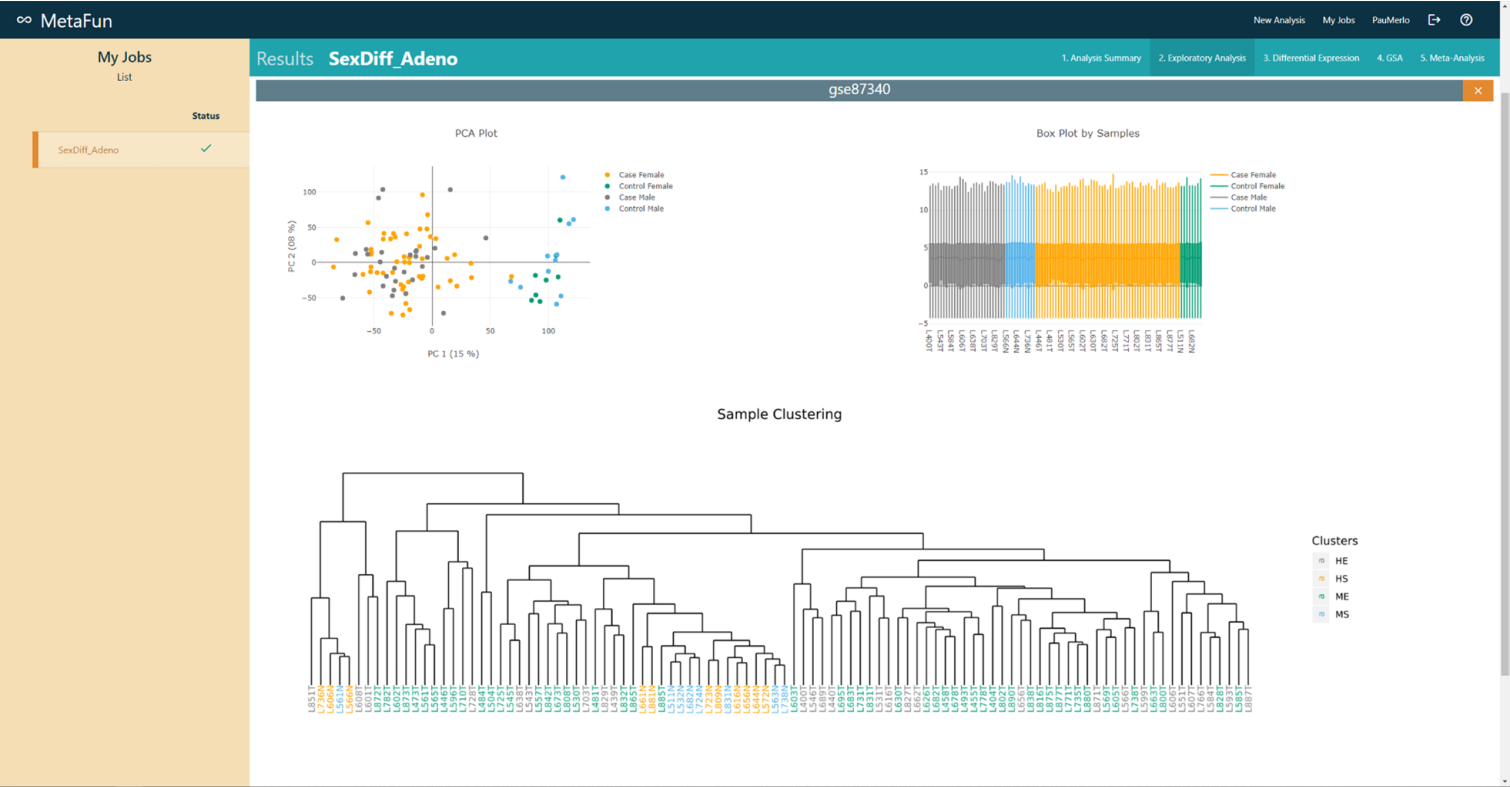


#### 3. Differential expression

The identification of genes showing differential expression by sex in affected patients for each study (Clicking on each link to the gene identifiers expands their biological information):
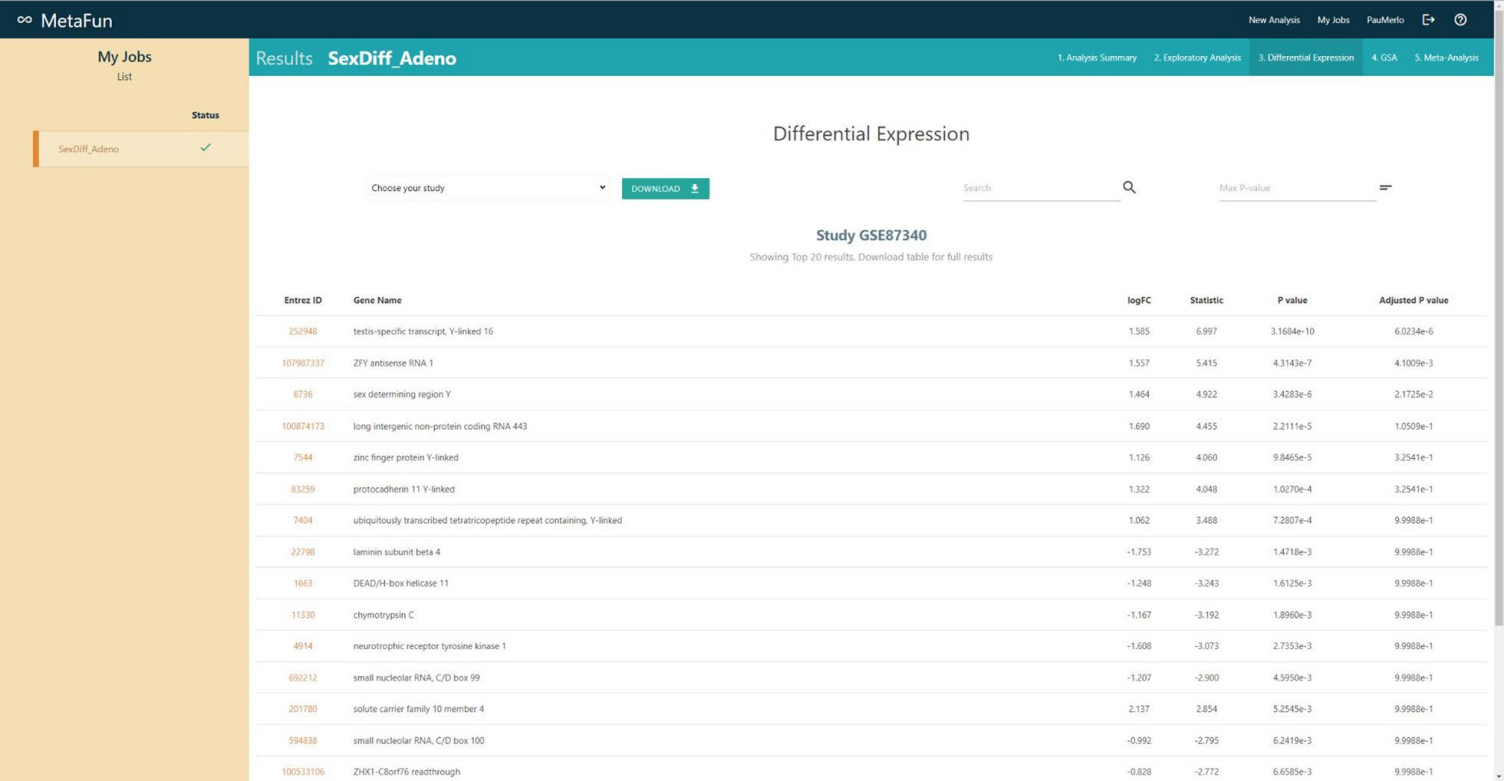


#### 4. Gene set analysis (GSA)

Functional characterization of the differential expression results identifies those functions more active in males and females (Clicking on each link to the identifiers expands the information for each significant function):
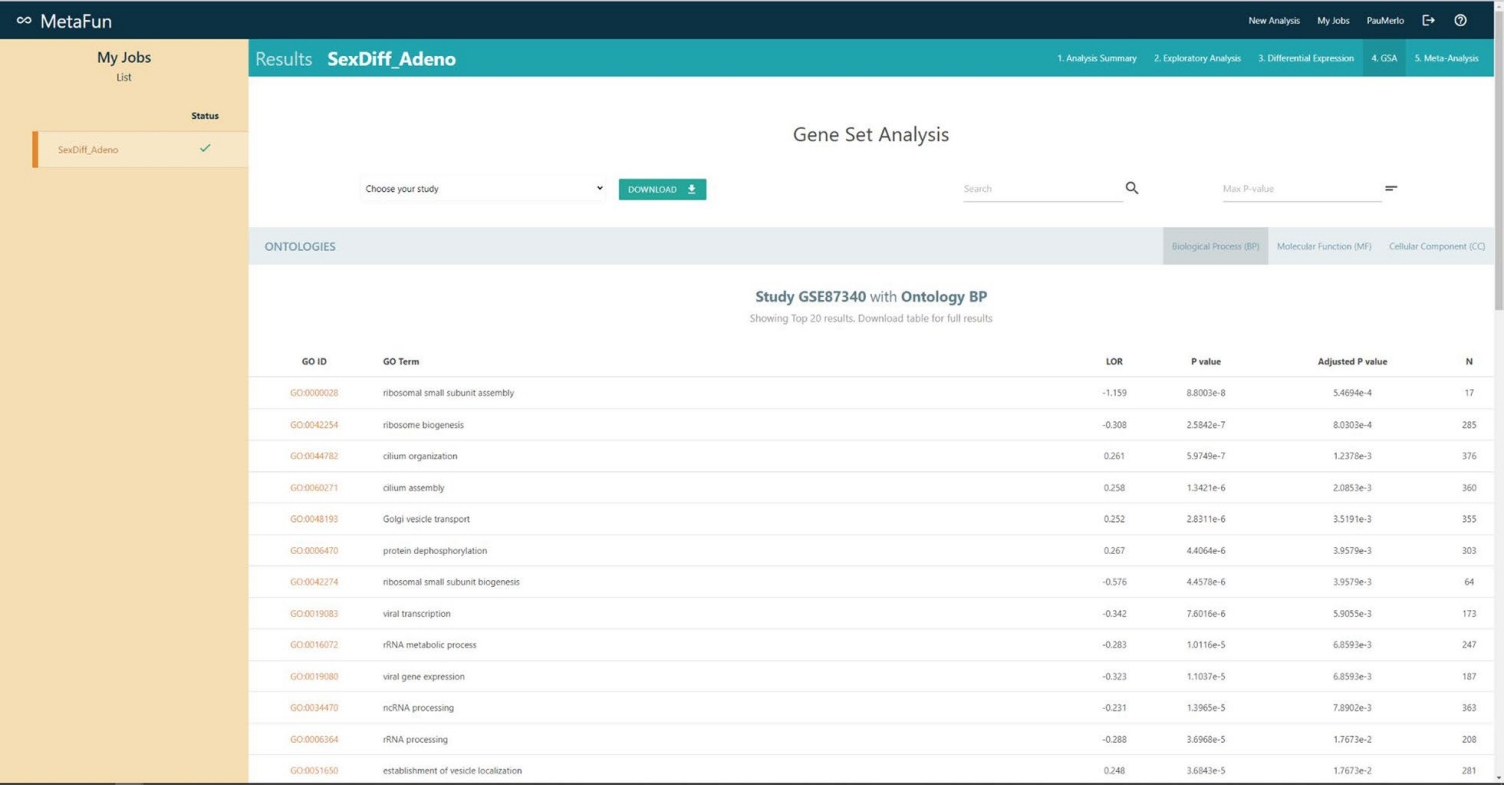


#### Meta-analysis

Demonstration of MetaFun functions and the pathways activated in evaluated studies (Clicking on the information icon leads to detailed information on these significant functions):
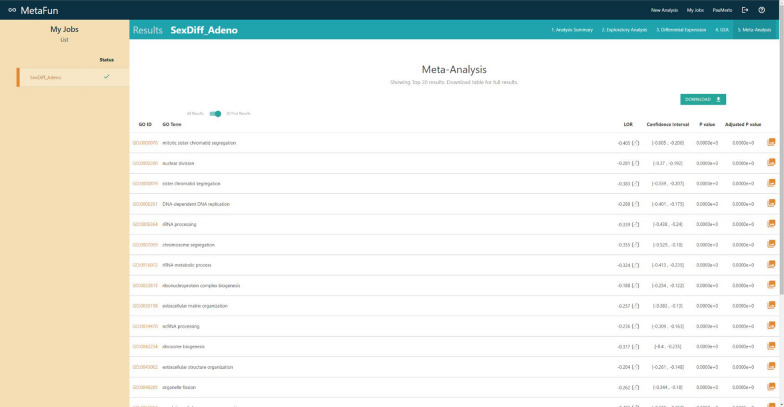



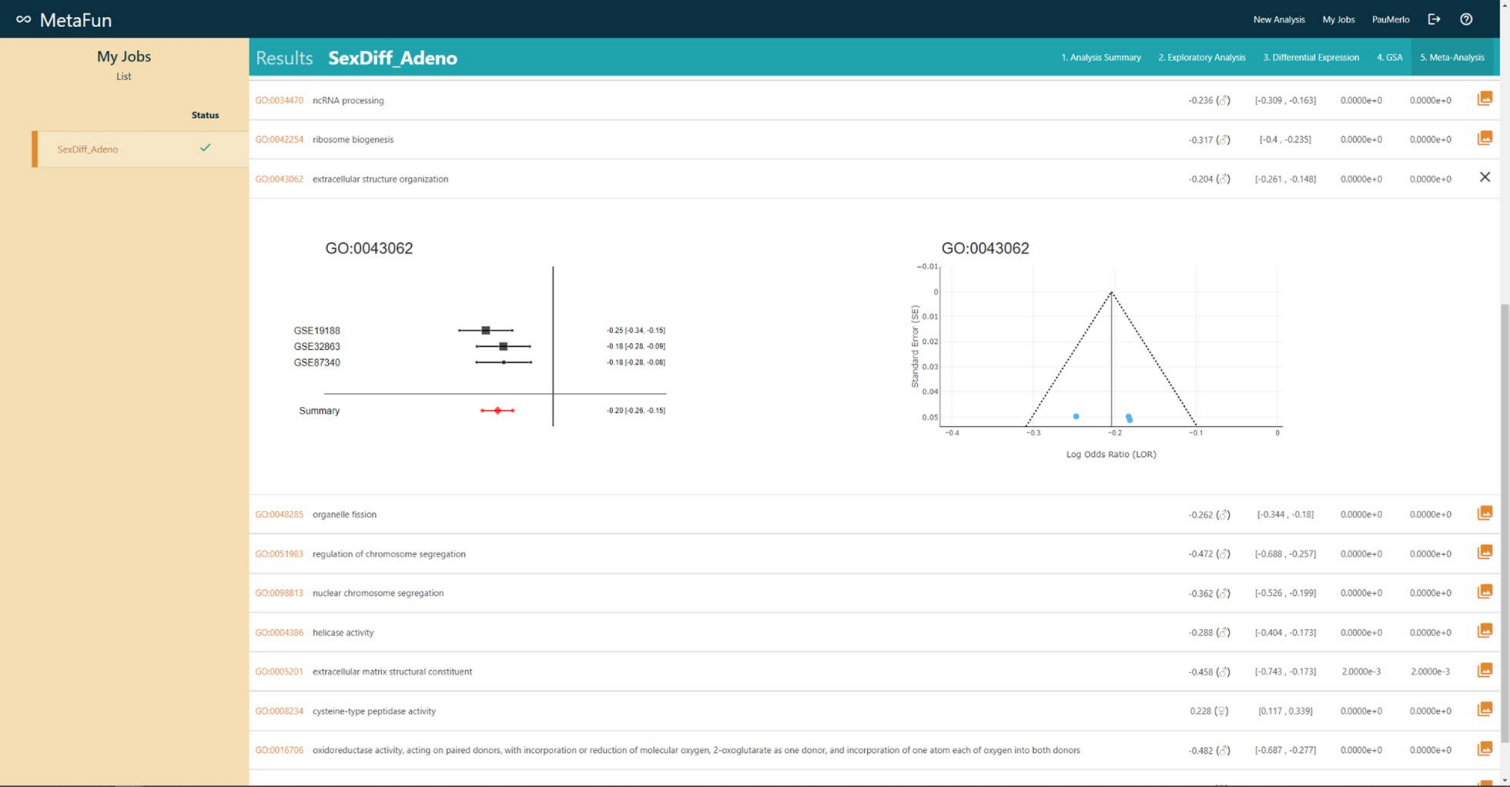


## Conclusions

Metafun (https://bioinfo.cipf.es/metafun) is a powerful and easy-to-use open-access web-based tool that analyzes and integrates multiple transcriptomic datasets to identify consensus biological functions to explain sex differences in numerous diseases. Its application will improve the incorporation of the sex perspective in biomedical studies, generating novel knowledge that can be useful in the application of an effective Personalized Medicine that includes the relevant information of the sex of the patients.

## Data Availability

MetaFun is freely available at http://bioinfo.cipf.es/metafun. The back end was implemented in R and Java, and the front end was developed using Angular. R code available at https://gitlab.com/ubb-cipf/metafunr.
