# Correction: MetaFun: unveiling sex-based differences in multiple transcriptomic studies through comprehensive functional meta-analysis

**DOI:** 10.1186/s13293-024-00646-8

**Published:** 2024-09-12

**Authors:** Pablo Malmierca-Merlo, Rubén Sánchez-Garcia, Rubén Grillo-Risco, Irene Pérez-Díez, José F. Català-Senent, María de la Iglesia-Vayá, Marta R. Hidalgo, Francisco Garcia-Garcia

**Affiliations:** 1 Computational Biomedicine Laboratory, Principe Felipe Research Center (CIPF), Eduardo Primo Yúfera Street, 3, Valencia, 46012 Spain; 2 Biomedical Imaging Unit FISABIOCIPF, Fundación Para el Fomento de la Investigación Sanitaria y Biomédica de la Comunidad Valenciana, Valencia, 46012 Spain; 3 Department of Mathematics, Faculty of Mathematics, University of Valencia (UV), BurjassotValencia, 46100 Spain

**Correction: Biol Sex Differ 15**,** 66 (2024)**

10.1186/s13293-024-00640-0

Following publication of the original article [1], the authors reported an error in the funding statement.

## Incorrect

This research was supported and partially funded by GV/2020/18, the Institute of Health Carlos III (project IMPaCT-Data, IMP/00019), co-funded by ERDF, “A way to make Europe”; PID2021-124430 A-I00 funded by MICIU/AEI /10.13039/501100011033 and by FEDER, UE.

## Correct

This research was supported and partially funded by GV/2020/18, the Institute of Health Carlos III (project IMPaCT-Data, IMP/00019), co-funded by ERDF, “A way to make Europe”; PID2021-124430OA-I00 funded by MICIU/AEI/10.13039/501100011033 and by FEDER, UE.

The original article [1] has been corrected.
